# Sensitivity Evaluation for Global Perturbations in Non‐Hermitian Skin‐Effect Sensors

**DOI:** 10.1002/nap2.70039

**Published:** 2026-02-25

**Authors:** Letian Yu, Cesare Soci, Y. D. Chong, Baile Zhang

**Affiliations:** ^1^ Division of Physics and Applied Physics, School of Physical and Mathematical Sciences Nanyang Technological University Singapore; ^2^ Centre for Disruptive Photonic Technologies Nanyang Technological University Singapore

## Abstract

Non‐Hermiticity has introduced new physical mechanisms into sensing, with approaches based on exceptional points and non‐Hermitian skin effects demonstrating potential sensitivity enhancements over conventional sensing technologies. By monitoring the frequency shifts of specific eigenmodes, previous studies on non‐Hermitian sensors have revealed extraordinary sensitivity to local perturbations. In contrast, the influence of global perturbations such as noise and disorder, which generally involve complex spectra and may even suppress these eigenmodes, seems largely incompatible with the current non‐Hermitian sensing framework and has received far less attention. Here, motivated by recent theoretical advances on pseudospectra theory, we investigate the possibility of employing maximum transient growth to probe the level of global perturbations in non‐Hermitian skin‐effect sensors. Using discrete‐time light walks in synthetic photonic lattices, we experimentally evaluate the performance of a non‐Hermitian photonic lattice under static global phase noise. Remarkably, we demonstrate that the sensitivity grows exponentially with lattice size, manifesting in the maximum transient growth rather than the spectral shifts of previous non‐Hermitian skin‐effect sensors. Furthermore, numerical simulations reveal that this exponential sensitivity is preserved under dynamical perturbations. Our results highlight the limits as well as the potential of non‐Hermitian systems to tackle a wide range of sensing requirements for next‐generation ultrasensitive sensors.

## Graphical abstract

Non‐Hermitian sensing against local and global noise. Conventional non‐Hermitian sensors focus on sensitivity against a static local boundary defect. Our work extends that framework to detect global perturbations. Using synthetic photonic lattices, we experimentally verify that sensitivity to global static and dynamic disorder scales exponentially with system size, unlocking the potential for ultrasensitive detection of distributed fluctuations or noise.

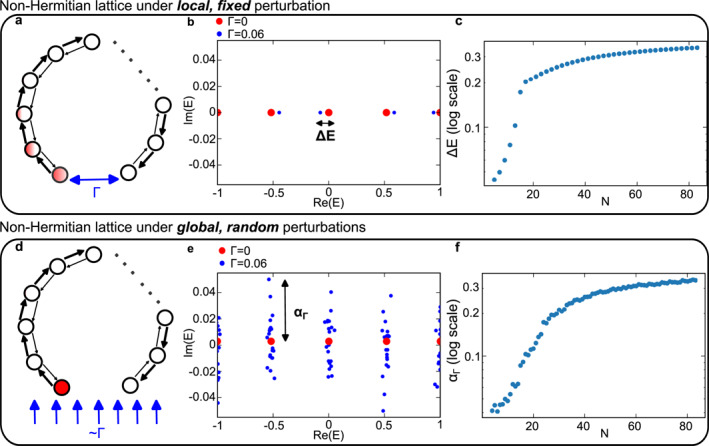

## 1 Introduction

Noise and disorder are ubiquitous, and both the extraction of weak signals from them and their precise characterization are critical to modern sensing technologies [1, 2, 3, 4, 5, 6, 7]. However, in most cases, an inherent trade‐off exists between sensitivity and noise resilience: Sensing systems that exhibit high susceptibility to the signal of interest also tend to be unstable against random fluctuations [8]. A typical example is a sensor operating at an exceptional point (EP) [9, 10, 11, 12]. For a small deviation ϵ away from the EP of order m, the energy splitting scales as $\Delta E\propto \epsilon^{1/m}$. This corresponds to a sensitivity that can be dramatically enhanced by increasing the order m. However, noise resilience, and more generally the precision of the sensor, deteriorates significantly due to the coalescence of modes at the EP [13, 14, 15, 16, 17, 18, 19, 20, 21, 22, 23]. In particular, the overall detector response, taking into account of the noise contributions, tends to show a net performance that is similar to its conventional counterpart [13, 14, 24].

**FIGURE 1:**
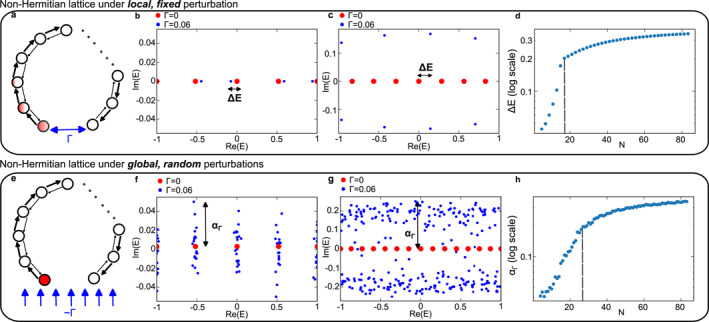
NHSE sensor under local and global disorders. (a) Schematic diagram of the sensors based on NHSE. The signal to be detected is labeled by the coupling Γ between two resonators. (b) Eigenvalue spectra of the lattices in (a) with Γ=0 and Γ=0.06. The lattice parameters are N=11, $J_{L,R}=e^{\pm 0.2}$. (c) Same as (b) but N=21. (d) Shift in the zero‐mode eigenvalue as the lattice size N increases. (e) NHSE sensor under global disorder, where the perturbation matrix has a matrix 2‐norm of Γ. (f) Eigenvalue spectra of the lattices in (c) with N=11, Γ=0 and Γ=0.06. The latter consists of 20 realizations. (g) Same as (f) but N=41. (h) The abscissa as the lattice size N increases.

An alternative paradigm is offered by sensors based on the non‐Hermitian skin effect (NHSE), which exploits the synergy between non‐Hermiticity and topology [25, 26, 27, 28, 29, 30] (Figure 1a). In such systems, the real frequency shifts of topological zero modes scale exponentially with increasing lattice size under boundary localized disorders, which act as local perturbations. The key idea behind this approach is that by measuring the spectrum shifts Re(ΔE) in response to the changes in end‐to‐end coupling Γ (lattice size N=11 in Figure 1b and N=21 in Figure 1c), one can determine the perturbation strength directly. Such arrangements provide two distinctive advantages: the sensitivity increases exponentially with lattice size up to a critical value (Figure 1d), whereas the response remains robust against random fluctuations within the underlying lattice that do not affect the boundary condition [25].

However, this framework breaks down when global disorder, instead of local perturbations, becomes dominant. Unlike localized disorder, which can be analyzed through individual eigenvalue shifts, global noises that naturally arise from pump fluctuations or other environmental factors tend to affect all lattice sites simultaneously, resulting in collective spectral perturbations. Furthermore, since the underlying spectrum for NHSE sensors is nonorthogonal, any small perturbation to the sensing mode can excite nearby modes, leading to nonlinear sensor responses or the breakdown of the NHSE [31]. Consequently, NHSE sensors are generally considered relevant only in the regime of small local perturbations.

Challenging this common belief, recent theoretical studies [32, 33, 34] have proposed a shift from conventional perturbation theory, which focuses on changes in a single eigenvector, to the pseudospectra framework [35] that captures the collective impact of perturbations on all eigenvectors for sensing against global perturbations [36, 37, 38]. Remarkably, exponential sensitivity similar to that of the NHSE sensor for local perturbations can be found through both spectra and power‐dynamics analysis when these disorders turn global [34]. A conceptual schematic of this pseudospectra approach is shown in Figure 1e, where the end‐to‐end coupling disorder in Figure 1a is replaced by a more general perturbation matrix form $H^{\prime }$ of the same strength in terms of the matrix 2‐norm $\|H^{\prime }\|=\Gamma$. Here, we show the results from 20 perturbed matrices of Γ=0.06 onto the original Hamiltonian H in Figure 1e and the associated spectra, also known as the pseudospectra, are shown as blue dots in Figure 1f,g, which corresponds to the collection of complex numbers satisfying

(1) $$\sigma_{\Gamma }\left(H\right)=\left\{z\in C:z\in \sigma \left(H+H^{\prime }\right),\|H^{\prime }\|\leq \Gamma \right\}.$$

Here, a metric for the extent of the pseudospectrum is provided by the pseudospectral abscissa $\alpha_{\Gamma }=\max\left(\text{Re}\;\left(\sigma_{\Gamma }\left(H\right)\right)\right)$, that gives a measure of the lattice's spectral sensitivity. Interestingly, here we found this abscissa demonstrates a similar exponential sensitivity (Figure 1h) to that observed for the topological zero mode energy shift in NHSE sensors (Figure 1d).

To experimentally access this spectral sensitivity, we rely on the connection between the pseudospectrum and the power dynamics of the system. Although the pseudospectral abscissa itself is hard to be accessed directly, the Kreiss Matrix theorem [33, 35] establishes a link between the pseudospectral abscissa and the maximum transient growth of the system:

(2) $$\sup_{\Gamma >0}\left(\alpha_{\Gamma }/\Gamma \right)\leq \sup_{t}P_{\max}\left[H\right]\left(t\right)\leq eN\sup_{\Gamma >0}\left(\alpha_{\Gamma }/\Gamma \right)$$

where $P\left(t\right)=\|e^{-iHt}\psi_{0}\|$, $P_{\max}\left[H\right]\left(t\right)$ denotes the maximum attainable field amplitude, e is the Euler's number and N is the system size. This inequality implies that a large spectral sensitivity inevitably leads to a large transient growth [34]. Therefore, a practical route to estimate the global perturbation strength Γ is by measuring the maximum field amplitude P(t) across multiple disorder realizations.

**FIGURE 2:**
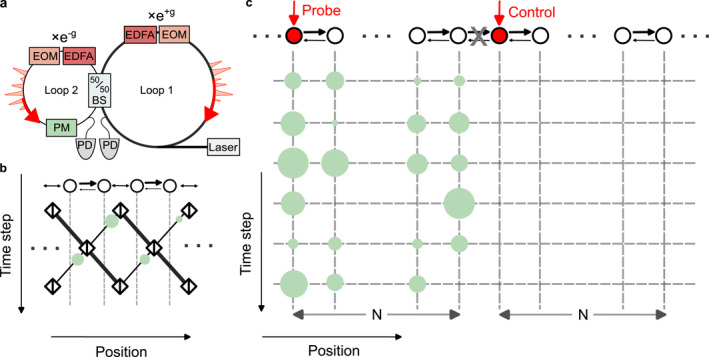
Schematics of the coupled fiber loop platform. (a) Simplified schematic diagram of the coupled fiber loops, where signals emitted by a continuous‐wave (CW) laser are shaped into square pulses of 100 ns width. (b) Mapping of the time‐bin encoded square pulses in (a) into a light walk. The relative gain and loss are represented by different shades of black bonds connecting individual synthetic lattice sites. (c) Schematic of the experimental procedures to study dynamic phase disorders. The probe and control lattices are separated as indicated by the cross in between them. Phase disorders of different strengths are represented by green shades of different sizes.

In this work, we numerically and experimentally employ this approach to study photonic quantum walks with global phase disorders [39]. Our platform is a coupled fiber‐loop system, where different time bins encode synthetic lattice positions and nonreciprocal hoppings are realized through intensity modulation [40]. Following the procedure outlined in Figure 2c, we prepare the initial excitation at the leftmost site of the NH lattice and measure the total intensity to find its correspondence with the perturbation strength in our system. Using non‐Hermitian lattices with sizes ranging from N=12 to N=20, we experimentally validate the increase of sensitivity under the same level of static phase disorders. Furthermore, our numerical simulations reveal that similar exponential sensitivity also arises in the presence of dynamic phase disorders in our system. These observations provide concrete evidence of the close connection between maximum transient growth and global perturbations in NH systems, highlighting their potential relevance for sensing applications.

## 2 Results

Here, we implement the non‐Hermitian lattice by introducing anisotropic hopping via gain and loss modulations for different loops as shown in Figure 2a, and the dynamics of the walk follow the following evolution equation.

(3) $$u_{n}^{m+1}=\cos\;\beta \left(u_{n+1}^{m}+i\;\sin\;\beta v_{n+1}^{m}\right)e^{-g+i\phi_{n}^{m+1}},$$

(4) $$v_{n}^{m+1}=i\;\sin\;\beta \left(iu_{n-1}^{m}+\cos\;\beta v_{n-1}^{m}\right)e^{g},$$

where $v_{n}^{m}$ corresponds to pulses propagating in Loop 1, and $u_{n}^{m}$ to those in Loop 2. Pulses in the two loops are coupled by a beamsplitter with a coupling coefficient β, which is set to be π/4 throughout the experiment. The strength of the anisotropic coupling is governed by the parameter g, controlled by an electrical‐optical modulator (EOM) together with an in‐loop erbium‐doped fiber amplifier (EDFA) that provides amplification in the long loop and attenuation in the short loop. Here, we take g=0.2 for all the experiment results shown subsequently. Phase noise, denoted as $\phi_{n}^{m}$, is implemented by a phase modulator (PM) in the short loop. Such arrangement effectively maps our setups to a Floquet 1D lattice model hosting anisotropic couplings as shown in Figure 2b, which has been studied previously to demonstrate NHSE [40]. Unlike earlier works [40, 41], here we use OBC at both ends of the 1D anisotropic lattice to tune lattice sizes accordingly.

In our experiments, in addition to the probe pulse, we also inject a control pulse into the neighboring unoccupied time slot, which remains uncoupled from the probe signal, as shown in Figure 2c. Here, no external phase modulation is applied to the control lattice. This comparison serves to accurately capture fluctuations in the EDFA as well as any long‐term disorder during the measurements. In the absence of perturbation, the control lattice corresponds to an NH‐SSH model under OBC. Using the control pulse intensity as a reference, we can then isolate the contribution of phase noise to variations in the overall intensity and obtain the corresponding maximum power amplitude $P_{\max}\left[H_{\Gamma }\right]$ through multiple measurements. Furthermore, the maximum power amplitude in the control lattice is also recorded as $P_{\text{ref}\;}=P_{\max}\left[H_{\Gamma =0}\right]$ for normalization purposes.

**FIGURE 3:**
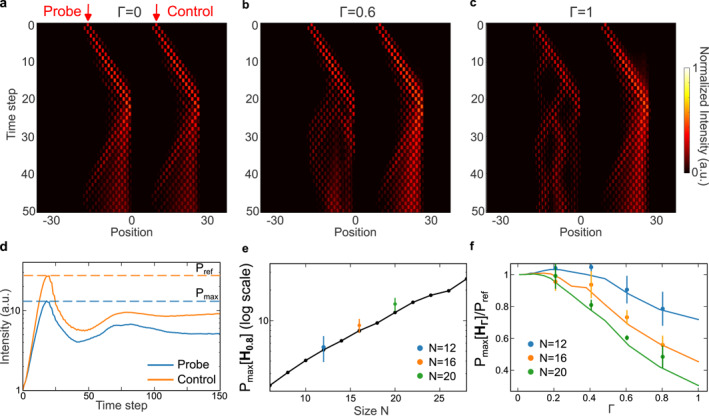
Experimental demonstration of evaluating sensitivity via maximum power amplitude. (a–c) Experimentally obtained time evolution showing the pulse dynamics under different levels of disorder for N=16. The control lattice is located on the right of the probe lattice in all three subplots. (d) Comparison of intensity between the probe and control pulses. Their respective maximum power amplitude is denoted by dashed lines. (e) Experimentally measured maximum amplification when Γ=0.8, for different lattice sizes N=12,16,20. Numerically computed results are shown as the black dots and the solid curve. (f) Experimentally measured shifts of the amplification ratio against the coupling strength Γ for different lattice sizes. Numerically calculated values are shown as solid curves.

We start our experiments by probing anisotropic lattices with static phase noise that remains unchanged across different time steps. Both the probe and control lattices are excited from the leftmost unit cell, which is farthest from the skin edge. The overall intensity evolution is then monitored repeatedly for different disorder realizations. The corresponding transport behaviors are shown in Figure 3a–c for different phase‐disorder strengths, with each providing a sequence of perturbations sampled from a uniform distribution [−Γπ,Γπ] and applied along the spatial index n. As the disorder strength increases, the non‐Hermitian skin effect is suppressed by Anderson localization, which traps the wavepacket near the excitation center through destructive interference, leading to a continuous crossover between the two localization mechanisms.

A typical intensity measurement result for a single disorder realization is shown in Figure 3d, where the maximum intensity levels reached in both lattices are indicated by dashed lines and used for subsequent sensitivity calculations. Considering 20 independent disorder realizations, we present the experimentally measured $P_{\max}\left[H_{\Gamma =0.8}\right]$ for anisotropic lattices of different sizes, together with the corresponding simulation results in Figure 3e. The data show a clear exponential relation between the maximum transient growth and the lattice size. To further characterize the sensor performance, we apply perturbations ranging from Γ=0.2 to Γ=0.8 for lattices of size N=12,16,20, respectively. As shown in Figure 3f, the experimental results are in good agreement with simulations. The deviation between $P_{\max}$ and $P_{\text{ref}\;}$ exhibits a clear increasing trend with both lattice size and perturbation strength.

Next, we investigate the sensitivity of our NH lattice in the presence of a dynamic phase disorder, a prominent source of disorder when systems are subjected to temporal fluctuations. Generally, dynamic phase disorders are known to be detrimental toward interference phenomena like Anderson localization, leading to decoherence and dephasing [42, 43, 44, 45, 46]. In contrast, recent studies have shown that counterintuitive phenomena such as enhanced memory effects and persistent skin effects could be found even in the fully incoherent regime [47, 48, 49, 50, 51]. Although it is well understood the interplay between uncorrelated disorder and non‐Hermiticity has a huge impact on the wavepacket's transport behaviors [44, 49], there have been few studies on its influence over the overall power dynamics and its relation to sensing up till now.

**FIGURE 4:**
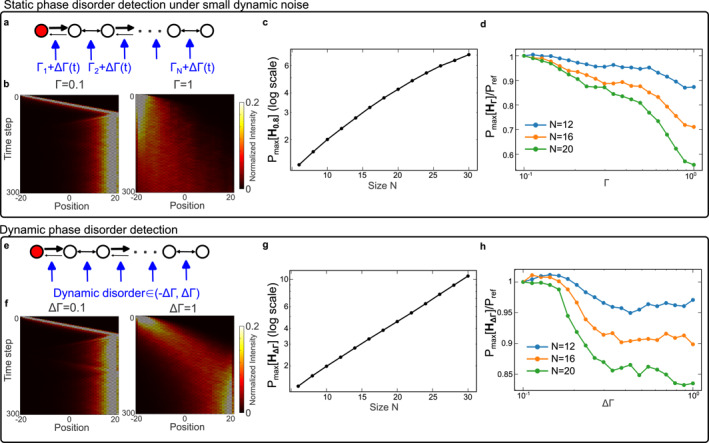
Numerical results for lattices under dynamic phase noises. (a) Schematics of the anisotropic lattices under static phase noise and an additional dynamic phase noise. (b) Simulated time evolution showing the pulse dynamics under different levels of the dynamic phase disorder for N=40 and g=0.1. The results are from the averages of 200 disorder realizations. (c) Numerically calculated maximum amplification when Γ=0.8 against different lattice sizes. (d) Numerically calculated normalized maximum power amplitude against the static perturbation strength Γ for different lattice sizes. (e)–(h) same as above, but for the case of purely dynamic phase noise.

In the first step, we start by introducing a small dynamic phase disorder and to see if the previous sensitivity against static disorder remains. To explore this, here we introduce an additional uniformly distributed phase disorder in both space n and time m from [−ΔΓπ,ΔΓπ] on top of a static phase disorder drawn from [−Γπ,Γπ] as shown in Figure 4a. In the limit of ΔΓ→1, this uncorrelated phase disorder turns the original quantum walk into a classical random walk [49] in which the evolution equation is now modified as

(5) $$\begin{array}{c} X_{n}^{m+1}=\left(\cos^{2}\;\beta X_{n+1}^{m}+\sin^{2}\;\beta Y_{n+1}^{m}\right)e^{-2g+2i\phi_{n}},\\ Y_{n}^{m+1}=\left(\sin^{2}X_{n-1}^{m}+\cos^{2}\;\beta Y_{n-1}^{m}\right)e^{2g} \end{array}$$

where $X_{n}^{m}=\bar{|u_{n}^{m}{|}^{2}},Y_{n}^{m}=\bar{|v_{n}^{m}{|}^{2}}$ denote the statistical average of the pulse amplitude at each site. Since such transition is driven purely by the presence of dynamic phase disorder, its impact over the system's dynamical sensitivity can be similarly probed using the pseudospectra theory. We start by considering the case in which ΔΓ=0.3 and the numerically obtained intensity evolution for lattices of different sizes is shown in Figure 4b. For small static disorder Γ=0.1, the transport behavior is dominated by skin‐mode localization, with all the light intensity directed toward the right boundary. This picture changes significantly when Γ=1. Anderson localization now takes over and the wavepacket again localizes close to the excitation site. In this case, both the maximum transient growth shown in Figure 4c and the sensitivity shown in Figure 4d follow nearly identical trends to those observed in the static case (Figure 3), suggesting negligible perturbations introduced by the small dynamical disorder.

Secondly, we proceed to study the NH lattice's sensitivity against pure dynamic disorder, where only dynamic phase disorder drawn from the uniform distribution [−ΔΓπ,ΔΓπ] is considered. In this case (Figure 4e), as ΔΓ increases, this system gradually approaches the fully incoherent limit (Equation 5) and finally corresponds to a classical random walk for incoherent light [43]. However, unlike the previous case where Anderson localization plays an antagonistic role against the skin effect, here the skin‐mode localization remains visible as the perturbation varies (Figure 4f). Consequently, although the maximum transient growth $P_{\max}\left[H_{\Delta \Gamma }\right]$ maintains its exponential dependence on the lattice size (Figure 4g), its ratio relative to the reference value $P_{\text{ref}\;}$ exhibits saturation once the disorder is sufficiently large to drive the system into the incoherent limit (Figure 4h). Overall, these results demonstrate that anisotropic lattices could achieve sensitivity enhancement even under dynamic perturbations. This contrasts with recent approaches based on Floquet engineering [30], where the lattice itself must be temporally modulated to enable the detection of dynamical perturbations.

**FIGURE 5:**
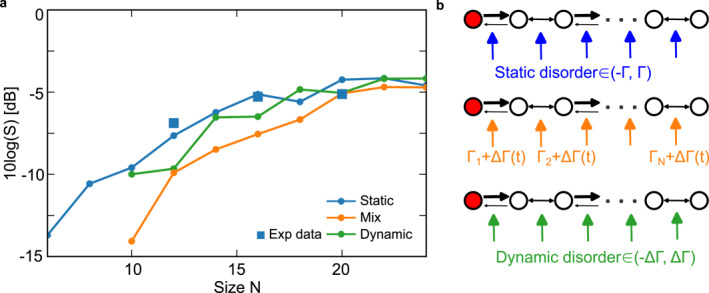
Exponential sensitivity in the non‐Hermitian sensors. (a) Numerically obtained sensitivities of the non‐Hermitian photonic systems with g=0.2 for different lattice size N (circles and solid curves). The experimentally obtained sensitivities are shown as blue squares. Here, three different types of global disorders are considered, which are depicted in (b) and correspond to the cases discussed in Figures 3 and 4. For the static and mix cases, Γ=0.6. For the dynamical case, ΔΓ=0.2.

Last, to quantitatively evaluate the performance of the sensors aforementioned, we calculate the sensitivity S=∂R/∂Γ, where $R=P_{\max}/P_{\text{ref}\;}$, as a function of the lattice size N both numerically and using our experimentally obtained data. The results are presented in Figure 5a. Clearly, the sensitivity of the non‐Hermitian systems exhibits a linear dependence on the lattice size, providing direct evidence of the exponential scaling relation $S\propto e^{\alpha N}$. This trend is consistent across different types of global disorders, regardless of purely static, dynamic or a mix of both (Figure 5b).

## 3 Discussion

In this work, we experimentally demonstrate the possibility of using maximum transient growth for sensitivity evaluation. For anisotropic photonic lattices subjected to varying levels of global phase disorder, we monitor the maximum intensity across multiple disorder realizations. The sensitivity clearly exhibits an increasing trend with lattice size and shows good agreement with numerical simulations. Finally, we further extend this approach toward dynamic noises' measurements, a feature generally believed to be incompatible with the static non‐Hermitian topological sensor [30]. Numerical results reveal that enhanced sensitivity persists even under dynamic perturbations and likewise scales with the system size.

Our findings shed light on the potential applicability of this intensity‐based sensing approach for advancing non‐Hermitian sensing devices. Since the underlying mathematical approach relies only on matrix analysis and is not restricted to this platform, we anticipate future studies will demonstrate similar effects in either acoustic settings, for which noises are generally more pronounced and globally distributed over the system [52], or the quantum regime [53], where a more comprehensive treatment including quantum Fisher information and quantum noise can be addressed. It would also be interesting to discuss the potential relevance with other non‐Hermitian phenomena such as scale‐free localization [54] and non‐Hermiticity‐induced topological phase transition [55] in this framework.

## 4 Experimental Procedure

To realize the probe and control lattices as shown in Figure 2, we start by generating two square pulses separated by ΔT=3.6μs from a CW laser source at 1550 nm wavelength, with their pulse widths being 100 ns. The two pulses are then injected into the coupled fiber loops, which have an average round trip time of 20μs and a time delay of 300 ns in between them. For pulses propagating in the longer Loop 1, their intensities are always amplified through a combination of EDFA and EOM. On the other hand, pulses in the shorter Loop 2 are always suppressed and are subjected to phase disorder fluctuations introduced by the phase modulator. The boundaries for probe and control lattices are implemented by EOM modulations as well, where perfect reflection at the OBC is modeled by introducing high suppression in the forward direction and the corresponding amplification in the backward direction.

## 5 Data Analysis and Processing

In our experiments, pulses at different time bins correspond to the intensities at different site numbers in the NH SSH lattice. For each disorder realization, the time traces are averaged over 200 instances and the square of the maximum intensity attained in the recorded data is recorded as 1 data point for the quantity $P_{\max}$. For each disorder strength in Figure 3, we repeat the procedure above for 20 different disorder realizations and the resulting distributions are reported in Figure 3f. We take the assumption that the results for different disorder realizations follow a normal distribution and derive the quantity $P_{\max}\left[H_{\Gamma }\right]=\text{mean}\;\left(P_{\max}\right)+\sqrt{2\;\ln\left(n_{\text{sample}\;}\right)\text{Var}\;\left(P_{\max}\right)}$. In addition, following the convention of other sensing schemes, we normalize the data point for all disordered realizations by the reference power $P_{\mathrm{ref}}$ that corresponds to the maximum power in the control group. In this case, when Γ=0, $P_{\max}\left[H_{\Gamma }\right]/P_{\text{ref}\;}=1$ for lattices of all sizes.

## Author Contributions

**Letian Yu:** methodology, investigation. **Cesare Soci:** writing – review and editing, supervision. **Y. D. Chong:** writing – review and editing, supervision. **Baile Zhang:** writing – review and editing, supervision.

## Supporting information


**Supporting Information:** nap270039‐sup‐0001‐suppl‐data.pdf.

## Data Availability

The data that support the findings of this study are available from the corresponding author upon reasonable request.
