# Supplementary material for: Sensitivity Evaluation for Global Perturbations in Non‐Hermitian Skin‐Effect Sensors
Source: Nanophotonics. 2026 Feb 25;15(5):e70039. doi: 10.1002/nap2.70039 (PMC12965027; doi:10.1002/nap2.70039)
Supplement: Supplementary file 1 — Supporting Information: nap270039‐sup‐0001‐suppl‐data.pdf. [file NAP2-15-e70039-s001.pdf]

Supplementary Materials for the Manuscript
"Sensitivity evaluation for global perturbations in non-Hermitian skin-effect sensors"

S1 Analysis using structured pseudospectra and general pseudospectra

The perturbation matrices discussed in Figure. 1d and Ref. [1] represent the general case in which all matrix elements are random. By contrast, in this study we focus on global phase disorder for the experiments and simulations presented in Figure. 3 and Figure. 4, where the perturbations only exist on the lower and upper diagonals of the NH lattice Hamiltonian. A different case would be that of Figure. 1a in the main text, where only a single matrix element is perturbed. While structured perturbations fall under a subset of general perturbations, eigenvalues under structured perturbations often provide limited information about the overall matrix behavior behavior and can vary differently with that from pseudospectra [2].

To test this, here we provide the associated pseudospectra (Figure. S1) and spectral sensitivity (Figure. S2) for all three cases discussed in this work, where perturbation matrices of different structures but same 2-norm are used. From Figure. S1 and Figure. S2, we can clearly see that when purely global phase disorder is concerned, the abscissa no longer scales exponentially with the system size and the sensitivity trend also differs completely with those of the other two cases.

In this case, the shape of initial excitation profile plays a more important role in the exponential sensitivity observed in the main text [3]. Here, we have chosen the excitation site to locate on the opposite side of the skin edge. Due to the non-reciprocity in the system, modes located there naturally propagate unidirectionally to the skin edge and are perturbed most. In contrast, modes located near the skin edge are less affected by the perturbation and as a result, their maximum transient growth varies less as they approaches the skin edge. In Figure. S3, we present the cases when the initial excitation shifts away from the leftmost unit cell. Since now the travelling distance between the excited modes and the skin edge decreases now, the transient time decreases which results in a lowered maximum amplification in general.

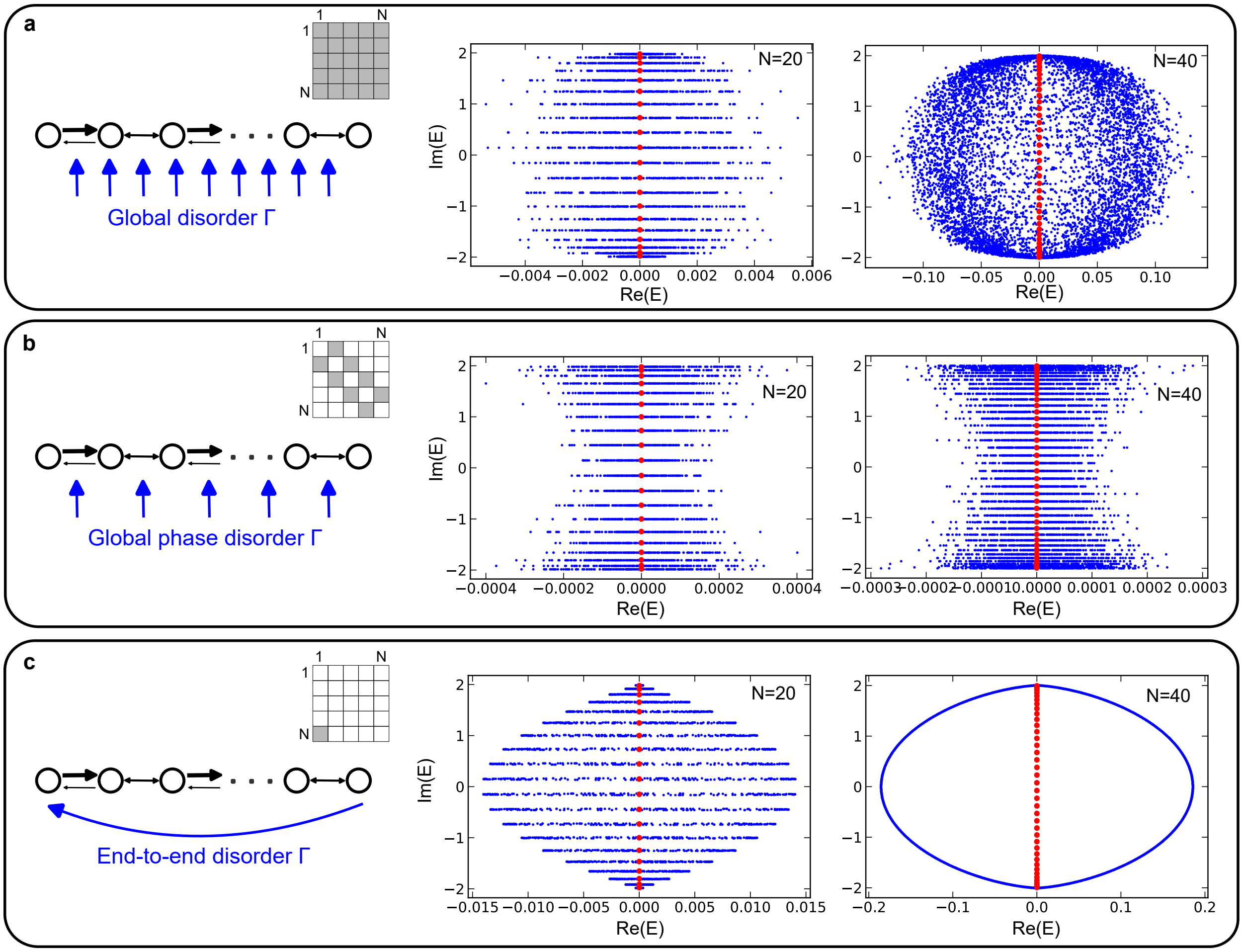

FIG. S1. **Pseudospectra for three different types of disorder implementations.** Complex spectra for NH-SSH lattices under three different types of disorder. In each case, non-zero elements of the perturbed matrix H' are indicated by the grey color and zero elements by white. We take $g = 0.5$ and compute 200 perturbed matrices for $\Gamma = 10^{-3}$ for each case.

S2 Hamiltonians for the Hatano-Nelson model in coherent and incoherent regimes

Here we provide the evolution equations describing the clean Hatano-Nelson model [4] in a 1D lattice with open boundary condition. The Hamiltonian and associated evolution equation are

$$\mathbf{H} = -\sum_{n=1}^{N-1} \left(J_L |n\rangle\langle n+1| + J_R |n+1\rangle\langle n| \right) \tag{S1}$$

$$\frac{d\psi}{dt} = -i\mathbf{H}\psi, \psi(t=0) = \psi_0 \tag{S2}$$

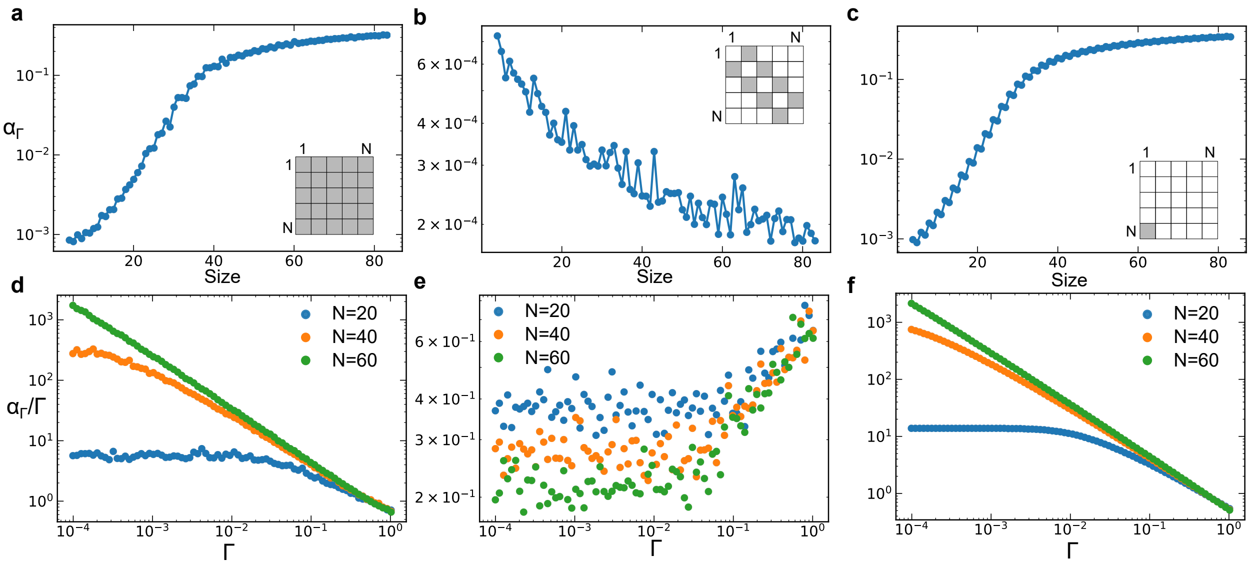

FIG. S2. **Scaling of spectral sensitivity for three different types of disorder implementations.** (a)-(c) variation of the structured pseudospectral abscissa α_Γ with size. (d)-(f) variation of sensitivity α_Γ/Γ with the disorder strength. We take $g = 0.5$ and compute 200 perturbed matrices for $\Gamma = 10^{-3}$ for a-c.

where the intitial condition ψ_0 is set to be the excitation located at the leftmost site $n = 1$ throughout. In this case, the solution of the wave equation turns out to be $\psi(t) = e^{-i\mathbf{H}t}\psi_0$ and the ratio $||\psi(t)||/||\psi(0)||$ corresponds to the square root of the intensity difference at these two moments.

The situation changes drastically when dynamical noises are considered, which wash out the phase information in the evolution process. While partial coherence dynamics are commonly analyzed by quantum master equations of Lindblad form, here for simplicity we approach this problem in the fully incoherent regime. In that case, the coherent dynamics are driven into the classical regime, in which transforms the phase randomization after each discrete time step changes the wavefunction picture $\psi(t)$ into an occupation probability description $P_n(t) = \overline{\psi_n^*(t)\psi_n(t)}$[5]. The governing master equation now becomes

$$M_{n,l} = 2\delta_{n,l}\ \mathrm{Im}\left(\mathbf{H}_{n,l}\right) + \Delta t\left[\left|\mathbf{H}_{n,l}\right|^2 - \delta_{n,l}\ \mathrm{Re}\left\{\sum_q \mathbf{H}_{n,q}\mathbf{H}_{q,l}\right\}\right] \tag{S3}$$

$$\frac{dP_n}{dt} = \sum_{l=1}^{N} M_{n,l}P_l(t) \tag{S4}$$

where $\mathbf{H}$ corresponds to the Hatano-Nelson model in the coherent limit.

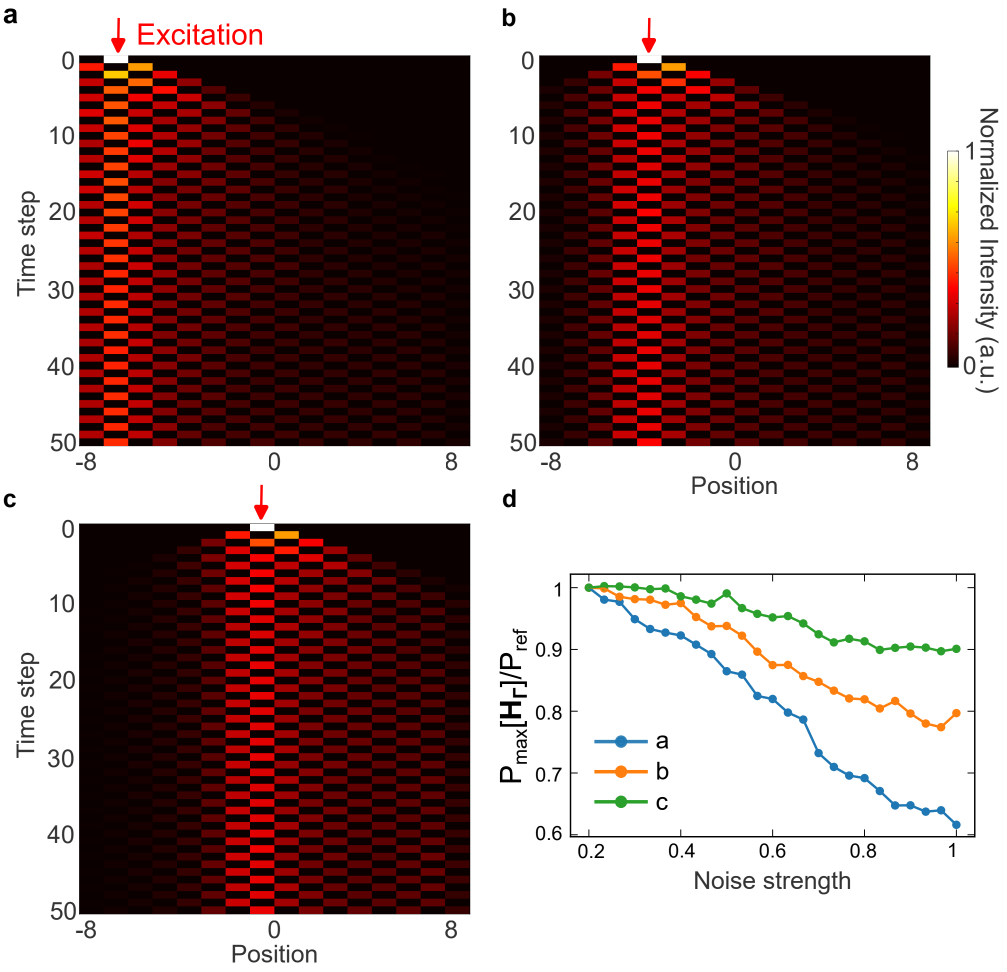

FIG. S3. **Dependence of maximum transient growth with initial excitation position.** (a)-(c) simulated time evolution showing the pulse dynamics under different initial excitation for $N = 16$ and $\Gamma = 1$. The results are from the averages of 200 disorder realizations. (d) variation of sensitivity with the disorder strength.

In the pseudospectra framework, for both coherent and incoherent cases we consider the collection of spectra for perturbed Hamiltonians with the following expression

$$\sigma_\Gamma^{\mathrm{OBC}}(\mathbf{H}) = \{z \in \mathbb{C} : z \in \sigma(\mathbf{H} + H'),\ \|H'\| \leq \Gamma\}\,. \tag{S5}$$

where $\lim_{\Gamma\to 0} \sigma_\Gamma^{OBC} = \sigma^{OBC}$ and the corresponding maximum singular value in the perturbed spectrum σ_Γ^{OBC} provides a good quantitative estimate for the maximum power amplitude following the Kreiss matrix theorem, as discussed in the main text.

Based on the discussions above, we calculate the complex pseudospectra in both cases and the results are shown in Figure S4. Here, the spectral sensitivity to perturbations

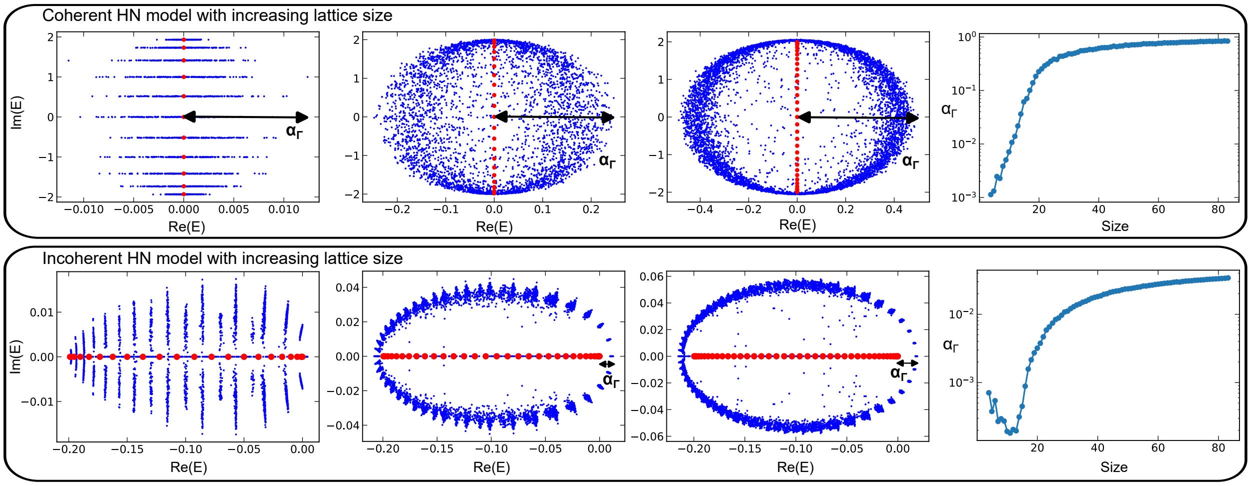

FIG. S4. **Schematic of complex pseudospectrum with increasing lattice size and fixed perturbation level.** As the lattice size N increases, the pseudospectrum (blue dots) turns from disk-clouds to a single cloud for both cases. Here, we use abscissa α_Γ, the maximum real value in each case, to characterize different pseudospectra. For both cases, the abscissa increases exponentially before certain threshold value. Here, we take $g = 0.5$ and compute 200 perturbed matrices for $\Gamma = 10^{-3}$ for each case.

is characterized by the abscissa $\alpha_\Gamma = \max \mathrm{Re}[\sigma_\Gamma^{OBC}]$. As shown in the rightmost panel of Figure. S4, in both cases we observe an exponential relation between the abscissa and the lattice size, similar to that being shown in Ref. [1].

---

[1] I. Kiorpelidis and K. G. Makris, Scaling of pseudospectra in exponentially sensitive lattices, Physical Review Research **7**, L032043 (2025).

[2] L. N. Trefethen, *Spectra and Pseudospectra: The Behavior of Nonnormal Matrices and Operators* (Princeton University Press, Princeton, 2005) description based on publisher supplied metadata and other sources.

[3] B. Midya, Topological directed amplification, Physical Review A **106**, 053513 (2022).

[4] N. Hatano, Localization Transitions in Non-Hermitian Quantum Mechanics, Physical Review Letters **77**, 570 (1996).

[5] S. Longhi, Incoherent non-Hermitian skin effect in photonic quantum walks, Light: Science & Applications **13**, 95 (2024)
